# An Aqueous-Based Approach for Fabrication of PVDF/MWCNT Porous Composites

**DOI:** 10.1038/s41598-017-01770-9

**Published:** 2017-05-11

**Authors:** Hossein Rezvantalab, Nastaran Ghazi, Matthew J. Ambrusch, Jeffrey Infante, Shahab Shojaei-Zadeh

**Affiliations:** Department of Mechanical and Aerospace Engineering, Rutgers, The state University of New Jersey, 98 Brett Road, Piscataway, New Jersey 08854-8058 United States

## Abstract

In this paper, we demonstrate the fabrication of conductive porous polymers based on foaming of an aqueous dispersion of polymeric particles and multi-walled carbon nanotubes (CNT). By tuning the surface energy of the constituents, we direct their preferential adsorption at the air-liquid (bubble) interface or within the liquid film between the bubbles. Sintering this bi-constituent foam yields solid closed-cell porous structure which can be electrically conductive if CNT are able to form a conductive path. We measure transport (electrical and thermal), mechanical, and morphological properties of such porous structures as a function of CNT loading and the method used for their surface functionalization. For a fixed polymer volume fraction, we demonstrate the limit in which increasing CNT results in decreasing the mechanical strength of the sample due to lack of adequate polymer-CNT bond. Such lightweight conductive porous composites are considered in applications including EMI shielding, electrostatic discharge protection, and electrets.

## Introduction

Porous polymer foams are conventionally produced using chemical/physical blowing agents or templating using droplets (emulsion) or solid particles (porogen)^1, 2^. Major limitations of these techniques include lack of control over pore size/size distribution, and applicability to only melt/solution-processable polymers^3–6^. High viscosity of polymer melts also proves to be challenging for uniform distribution of nanofillers in order to fabricate functional materials. Intractable polymers (*i.e*. those possessing a high glass transition temperature combined with a relatively low degradation temperature or those having a high molecular weight) cannot be easily melt-processed due to their high melt viscosity. However, such polymers are being used in many practical applications. For instance, Poly(ether ether ketone) (PEEK) is employed for its erosion resistance and excellent mechanical properties at elevated temperatures in pump and valve components as well as in transformer housings, battery holders, and fuel-line brackets. Poly(tetrafluoroethylene) (PTFE) is another example of an intractable polymer known for its non-reactivity and low friction properties in automotive industry. To fabricate porous materials from such polymers, different processes have been developed. They include dispersing of intractable polymer particles in thermally-stable matrices, sintering of uncompressed polymer powders, and inclusion of hollow microspheres typically made of glass, metal, and ceramics inside polymer matrices, *i.e*. syntactic foams^7–9^. However, these processes generally lead to low porosity (<40% air content) which may not be adequate for some applications^10, 11^.

An alternative approach to fabricate porous structures is to utilize particle-stabilized aqueous foams as precursor^11^. The process is based on the fact that fine solid particles can stabilize liquid-air interfaces resulting in foams that resist bubble coalescence and coarsening. Both melt-processable and intractable polymer particles can be used in this method provided that they have proper size and wetting properties. Foams stabilized by particles are reported to be less susceptible to coalescence, disproportionation, and coarsening in comparison to surfactant foams^12, 13^, allowing them to retain their microstructure for further processing^14–17^.

This approach can be extended to fabricate functional porous polymers by stabilizing aqueous foams using two constituents. One interesting feature of this method is that it enables controlling the positioning of the two components with respect to air-liquid interfaces^16^. Conductive nanofillers provide the opportunity to add functionality to insulating polymer foams without sacrificing mechanical properties^18, 19^. Low-density conductive polymers are of interest for use in electronic devices, especially those in aircraft, spacecraft, and automobiles *e.g*. for electromagnetic interference (EMI) shielding and electrostatic discharge dissipation^20–30^. Intrinsically conductive polymers exhibit poor processability and mechanical properties^31^. Alternatively, conductive polymers can be fabricated using conductive fillers such as carbon black, graphene, and carbon nanotubes^32–36^. Overlapping of the fillers throughout the structure may form a conduction path, leading to its electrical conductivity.

In this study, we prepare closed-cell conductive porous polymers with controlled porosity by incorporating multi-walled carbon nanotubes (CNT) into aqueous foams stabilized by polymeric poly(vinylidene fluoride) (PVDF) particles. The process involves preparing an aqueous suspension of PVDF particles and carbon nanotubes. In the first step, the CNT are functionalized for two main reasons: (1) to facilitate their uniform dispersion in the aqueous solution as their agglomeration deteriorates the mechanical properties of the resulting polymer structure, (2) to tune their hydrophobicity in order to direct their location with respect to the interface. We investigate two different methods to achieve this: covalent functionalization and non-covalent surfactant treatment^37, 38^.

Polymer particles are then added to the aqueous dispersion of CNT. The combined suspension is then mechanically frothed (air entrapment) using a mixer^39, 40^. The precursor aqueous foam is then dried at room temperature for several hours (to extract the excess liquid) followed by consolidation via sintering above the polymer melting point, forming solid porous structures^10^. We then investigate the effect of CNT concentration and functionalization method on morphology, density, mechanical, and transport (electrical, thermal) properties of the prepared porous nanocomposites.

## Experimental

### Materials

Preparing aqueous foams can be achieved using any type of polymer particles provided that they have the proper size and contact angle^10, 11^. In this work, we used commercially available poly(vinylidene fluoride) (PVDF) nanoparticles (Polysciences Inc.) with the primary particle size of *D*_*50*_ = 250 ± 30 *nm* and a contact angle of *~95°*, density of *1.76* *g/cm* ^3^, and melting point of *~160* *°C* ^10^. Ethanol was used to lower the surface tension and facilitate foaming and adsorption of particles to air-liquid (bubble) interfaces^10^. We used multi-walled carbon nanotubes with purity of >*90%* (Sigma-Aldrich) as conductive nanofillers. The reported average diameter of the CNT is *110–170* *nm* and their length is in the range of *10–30* *μm*. It should be noted that the nanofiller can be alternatively considered as carbon nanofiber noting the *10* ^*2*^ *nm* range of diameter, but the graphene sheet stacking and reported morphology is more similar to the nanotubes and we use this terminology throughout the text. The covalent functionalization of CNT was achieved using poly(vinyl alcohol) (PVA), 98% hydrolyzed with an average molecular weight of *M*_*w*_ = *13,000–23,000* (Sigma-Aldrich). PVA exhibits particularly strong interaction with CNT compared to other polymers such as poly(vinyl pyrrolidone) (PVP), and can crystallize at its surface and improve mechanical stress transfer^41^. Alternatively, we functionalized CNT with Triton X-100 (C_14_H_22_O(C_2_H_4_O)_n_), a non-ionic surfactant with critical micelle concentration (CMC) of 0.24 mM (0.0155%, w/v). This surfactant has been employed to aid in uniform dispersion of CNT in both polymer melts and aqueous solutions^42–47^.

### Foam production

**Figure 1:**
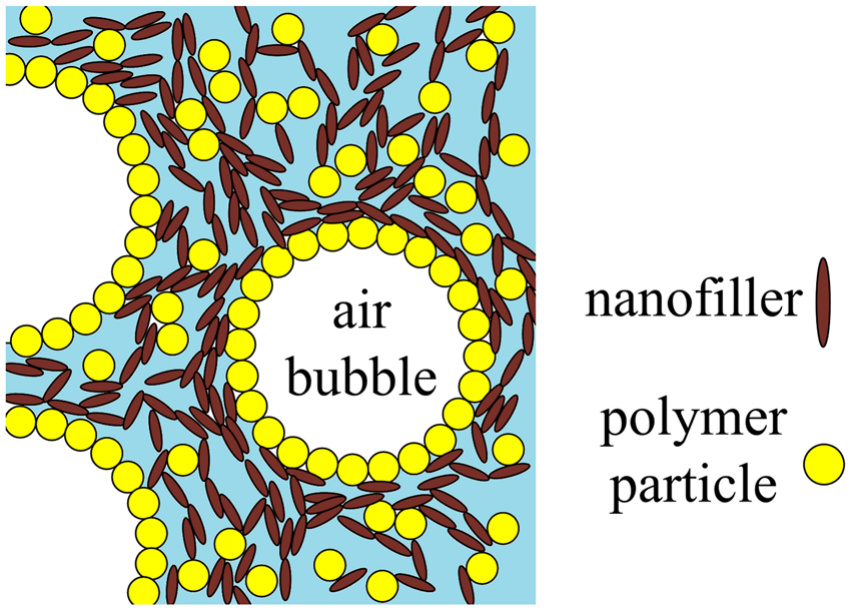
Schematic of the preferential adsorption of a binary system of polymeric particles and nanofillers to the liquid-air interface.

Particles with proper wettability can stabilize foams since their attachment at the air-liquid interface is energetically-favorable. Tuning particle surface energy (hydrophobicity) can lead to preferred adsorption of one component to the interface, as shown in Fig. 1. In this case, the hydrophilic nanofiller would prefer to remain in the liquid phase, and may form an inter-connected network upon consolidating the foam precursor.

PVDF nanoparticles were mixed with a 9% v/v solution of ethanol to lower the surface tension, facilitate foaming, and reduce the three-phase contact angle of the particles to the range 70°–74° suitable for foam stabilization^10^. The concentration of polymer particles was held fixed at 4% v/v throughout this work. To functionalize CNT, PVA was mixed with deionized water at a 5:10,000 (w/v) ratio, and the solution was heated at 70 °C for 2 hours under constant stirring. The desired CNT concentration (between 0–2 wt% with respect to water) was added to this heated solution and then treated in an ultrasonic bath for 30 min to ensure uniform dispersion of CNT. The dispersion was cooled overnight as functionalization happens during cooling^48^. Before adding polymer particles, the CNT aqueous solution was sonicated using Qsonica 500 W ultrasonic processor for 1 min at 50% amplitude using a 1/8″ tapered microtip. We expect the wettability of CNT to reduce from 110° to ~28° via this surface functionalization method based on the values previously reported in the literature^37^. In case of surfactant-treatment approach, an aqueous solution of Triton X-100 on a 5:10,000 (w/v) basis was prepared and stirred for 2 hours at room temperature. The desired CNT concentration was then added, followed by similar ultrasonic treatment to yield a homogeneous dispersion.

Subsequently, polymer particles were added to the aqueous solution of functionalized CNT, and probe-sonicated for 1 min to achieve efficient particle dispersion. Mechanical frothing of this suspension for 3–5 min using a mixer formed aqueous foam in which the bubbles are expected to be stabilized by PVDF particles, while functionalized CNT (with lower wettability) should remain in the thin liquid film between the bubbles. The prepared wet foam was then shaped into cylindrical molds and allowed to dry for 12 hours at ambient conditions to remove the excess water by gravity. After drying, samples were carefully taken out of the mold as they maintained a cylindrical shape and placed on an aluminum foil to reduce the effects of thermal gradients in vertical direction during sintering^31^. Samples were placed in an air convection oven and were heated gradually starting at 65 °C, with 10 °C increments every 10 minutes, till 165 °C. The samples were kept at this temperature (above polymer’s melting point) for 3 hours, after which consolidated porous structures were formed.

### Characterization

We used Scanning Electron Microscopy (SEM) to examine the morphology and porosity of sintered porous structures and the distribution of CNT within the foam matrix. Samples were freeze-fractured by dipping them in liquid nitrogen for 1 minute and then snapping them to get a sharp interface. They were then placed on a stud and gently attached to a carbon tape. To ensure the conductivity of the samples, they were coated with 25 nm of thermally evaporated gold before insertion into the vacuum chamber. We verified that thinner gold layers of even ~5 nm thickness could also deliver the electrical conductivity necessary for imaging, but used the default machine setting for the presented results. The porous morphology was then analyzed using Zeiss Sigma Field Emission SEM instrument.

The pore size distribution in porous structures is generally measured directly by inspecting the sample cross-section^49^. A procedure known as the line intercept method is described in the ASTM D3576 standard for this purpose. This method relies on drawing several lines across micrographs of the sample and counting the number of cell walls that intersect the reference lines. The length of the reference line should be large enough compared to the cell size being measured (typically crossing 10–20 cells). The average cell chord length *t* can then be determined by dividing the length of the reference line by the number of cells counted. It can be mathematically shown that the cell diameter can be calculated as *D* = *1.623* × *t* ^49^. By averaging the cell size calculated using several line segments/images, a reliable value can be obtained. This approach has been also used in earlier studies concerning composite foams^50–53^.

The apparent density of the porous composite can be calculated directly from the mass and geometric volume of the specimen. The porosity (void fraction) is obtained as *ϕ* = *1* *−* *ρ*_*f*_/*ρ*_*m*_, where *ρ*_*f*_ and *ρ*_*m*_ are the foam and matrix (polymer + CNT) density, respectively^54^. For mechanical characterization, we recorded the response of cylindrical samples ~17 mm in diameter and 13 mm in length to compressive loading using a universal testing machine (Instron Model 4411). The specimens were compressed between two smooth parallel plates with a compression rate of 1 mm/min according to ASTM D3574 standard for flexible cellular materials. The Young modulus was then extracted from the slope of the linear region in stress-strain curves.

To measure the electrical conductivity of the samples, flash-dry silver paint (Spi Supplies, Model# 04998-AB) was coated on top and bottom of cylindrical specimens to ensure good contact with electrodes. A two-probe method was used to measure the volume resistivity of the specimens with a high resistivity meter (Keithley Instruments, Model 2015). The measured resistance *R[Ω]* for a sample of cross-sectional area *A* and length *L* can be correlated to electrical conductivity *σ[S/m]* as *σ* = *L/RA*. Thin disc-shaped samples ~13 mm diameter and 1.5 mm thick were used to measure thermal diffusivity *α[m* ^*2*^ */s]* of porous composites using laser flash apparatus (NETZSCH NanoFlash System). The flash energy strikes the front surface of the sample creating a heat pulse, and thermal diffusivity is obtained using the time that it takes for the heat to travel through the sample and cause the temperature to rise on the other face. The reproducibility of the measured data was verified by firing the flash lamp several times and ensuring negligible deviation in the calculated diffusivity. Using the theoretical values of the heat capacity for PVDF and CNT from the literature, that of the composite was estimated based on their respective volume fraction: ${C}_{p}=\sum _{i}{(\varphi \rho {c}_{p})}_{i}$ ^55, 56^, where *i* refers to the two constituents and the volumetric heat capacity is ${C}_{p}=\rho {c}_{p}$. The thermal conductivity *k[W/m . K]* was then determined as ${k}_{c}=\alpha {C}_{p}$ for the porous nanocomposite.

## Results and Discussion

We fabricated specimens with CNT concentration in the range of 0–2 wt% in the aqueous solution and report the influence of filler concentration and functionalization method on their morphological, mechanical, and transport properties.

### Morphology and pore size distribution

**Figure 2:**
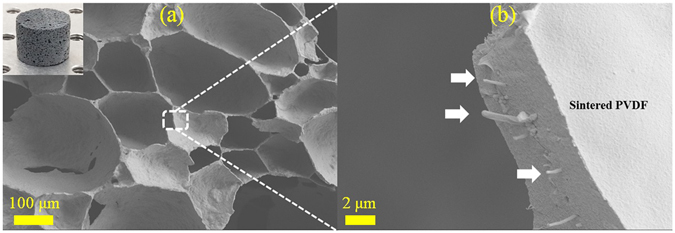
SEM micrographs showing (**a**) closed-cell pore structure and (**b**) CNT distribution in cell walls, for a typical PVDF/CNT porous composite.

Figure 2(a) shows SEM micrograph of cellular structure for a typical PVA-functionalized CNT-incorporated porous composite (the cylindrical sample is also shown in the inset). The microstructure reveals the formation of closed-cell pores. The pore-size varies due to the nature of frothing as well as the possibility of bubble coalescence leading to pores of various sizes.

Figure 2(b) is a close-up view of the sample, demonstrating the distribution of CNT along cell walls. We observe that it is possible to direct the conductive fillers to position between air bubbles by adjusting their wetting properties relative to polymer particles. Consequently, an inter-connected network may be formed throughout the matrix with sufficient CNT concentration, rendering the porous composite electrically conductive.

**Figure 3:**
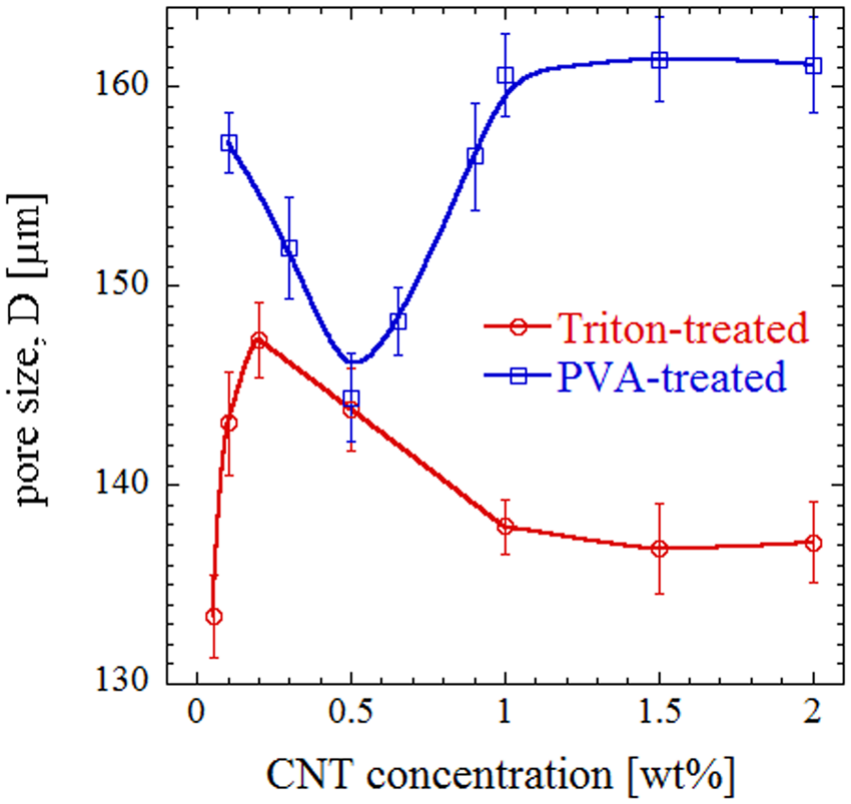
The average pore size of porous samples as a function of CNT concentration for the two functionalization methods.

For each composition, several SEM micrographs with 20X magnification were captured, and the line intercept method was applied in order to evaluate the average pore size. This yielded an average over more than 500 pores for each specimen, providing reasonable statistics according to the published standards^57^. The average pore size is plotted in Fig. 3 as a function of CNT concentration for two functionalization methods. The error bars are calculated based on the standard deviation of measured size for each line segment. We observe that for surfactant-treated CNT, the average pore size first increases up to 0.2 wt%, followed by a gradual decrease towards 1 wt% concentration of nanofillers, after which it becomes almost constant. The average pore size for samples with 2 wt% CNT is ~137 *μm*.

On the other hand, composite foams fabricated based on PVA-functionalization approach show a reduction in pore size up to 0.5 wt%, and then an increasing trend up 1 wt% CNT concentration, after which they approach a plateau of ~161 *μm*. This shows about 17.5% increase in average pore size in comparison with samples prepared based on similar CNT loading but using the surfactant treatment approach.

Both methods yield negligible change in pore size for CNT concentrations >1 wt%, which is possibly due to saturation of the porous structure with nanofillers and the inability of excess CNT to influence the microstructure. However, there is a disparity between the pore size trends at lower CNT concentrations based on the two functionalization methods, which may be due to interface stabilization when having surfactant molecules in the aqueous solution. We also note that PVA-treatment generally results in foams with larger pore size at similar CNT concentrations.

### Density and porosity

To characterize the apparent density, cylindrical samples of different CNT concentration are weighed on a precision balance. The apparent volume *V* is determined based on the measured diameter and height, and the density is calculated as *ρ*_*f*_ = *m*_*f*_ */V*.

**Figure 4:**
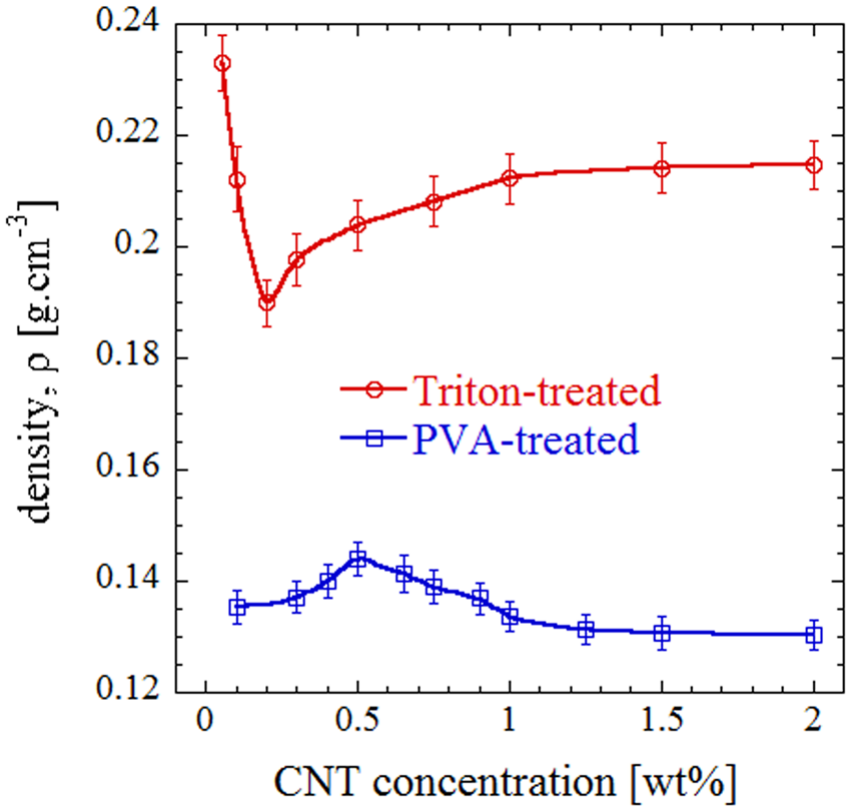
Density variation of porous samples as a function of CNT concentration for the two functionalization methods.

The density variation with CNT concentration in porous composites for both functionalization methods is plotted in Fig. 4. As expected, the trends are reversed in comparison with the pore sizes shown in Fig. 3. For Triton-treated samples, the density first drops by increasing CNT concentration up to 0.2 wt%, followed by a gradual increase above this minimum density point. On the other hand, PVA-treated specimens show an increasing density up to 0.5 wt% CNT concentration (which is the point corresponding to minimum average pore size of ~146 *μm*), and then a reduction at higher concentrations. The density becomes almost constant for samples prepared using both functionalization methods, approaching 0.215 g/cm3 and 0.130 g/cm3 for surfactant-treated and PVA-treated samples, respectively.

This shows that using PVA, the density can be reduced by 40% at higher CNT concentrations. This is generally consistent with their smaller average pore size observed in Fig. 3 based on the image processing of SEM micrographs. An increased pore size can be translated into reduced pore density, increased porosity, and thus reduced density of the resulting samples. However, the relative change in density appears to be stronger than the variation in pore size. We believe that this might be due to the difference in thickness of cell walls and concentration of PVDF particles per unit volume as their distribution and positioning in the sintered structure are influenced by the functionalizing method.

The relative density which is defined as the ratio of foam density to that of the matrix (solid polymer + filler), *ρ*_*f*_ */ρ*_*m*_ is typically used for comparing the air content of porous structures. The fabricated composites enable achieving relative densities as low as 0.074, which is much smaller than the 0.2 criteria set for low-density foams, and ~70% lower than the value reported for typical CNT-filled polymer foams^19, 58^. The maximum relative density is about 0.13, which is within the established range for light-weight materials^58^. The porosity (void fraction) calculated as *ϕ* = *1* − *ρ*_*f*_ */ρ*_*m*_^54^ is found to be in the range *86.8–92.6%*, which is considerably higher than the *40%* range reported by conventional foam fabrication methods^7–9^.

### Mechanical properties

**Figure 5:**
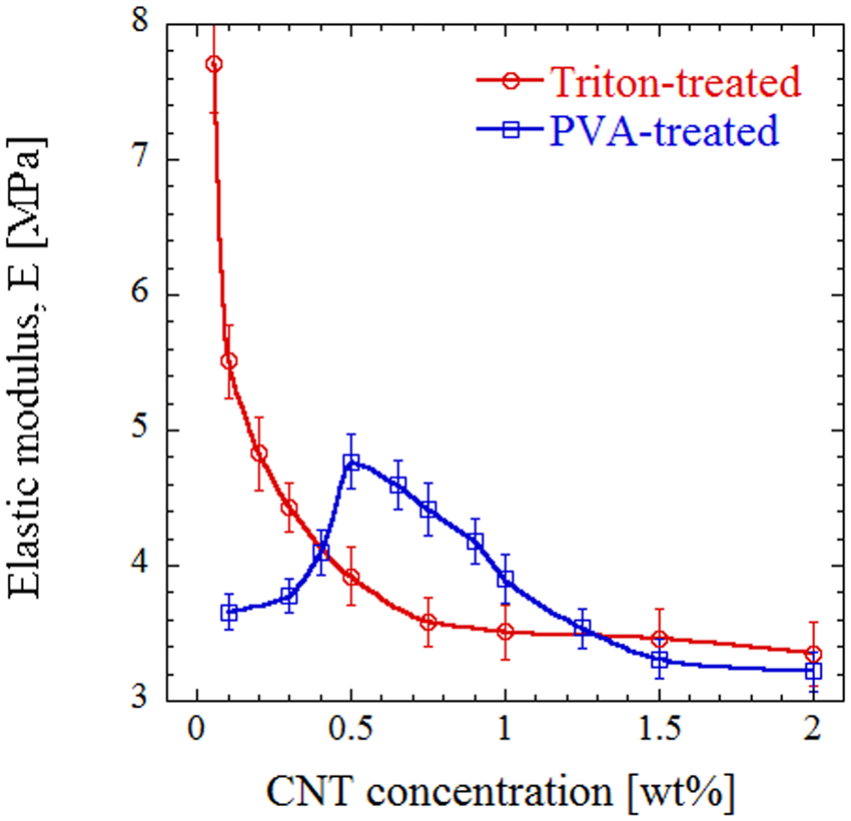
Compressive elastic modulus of porous samples as a function of CNT concentration for the two functionalization methods.

We compare the compressive elastic modulus of different specimens to characterize their mechanical properties. Figure 5 shows the elastic modulus as a function of filler concentration and functionalization method. We observe that incorporating PVA-treated CNT result in an increase in mechanical properties up to 0.5 wt% filler concentration, which corresponds to the maximum density point, namely 0.05 wt% for surfactant-treated case and 0.5 wt% for PVA-functionalization method. The modulus reduces as more CNT are incorporated into the polymer matrix possibly due to ineffective stress transfer between CNT and the polymer matrix. Although surfactant-treated samples show even higher modulii at filler loadings of <0.3 wt%, the mechanical properties deteriorates with incorporating more CNT.

**Figure 6:**
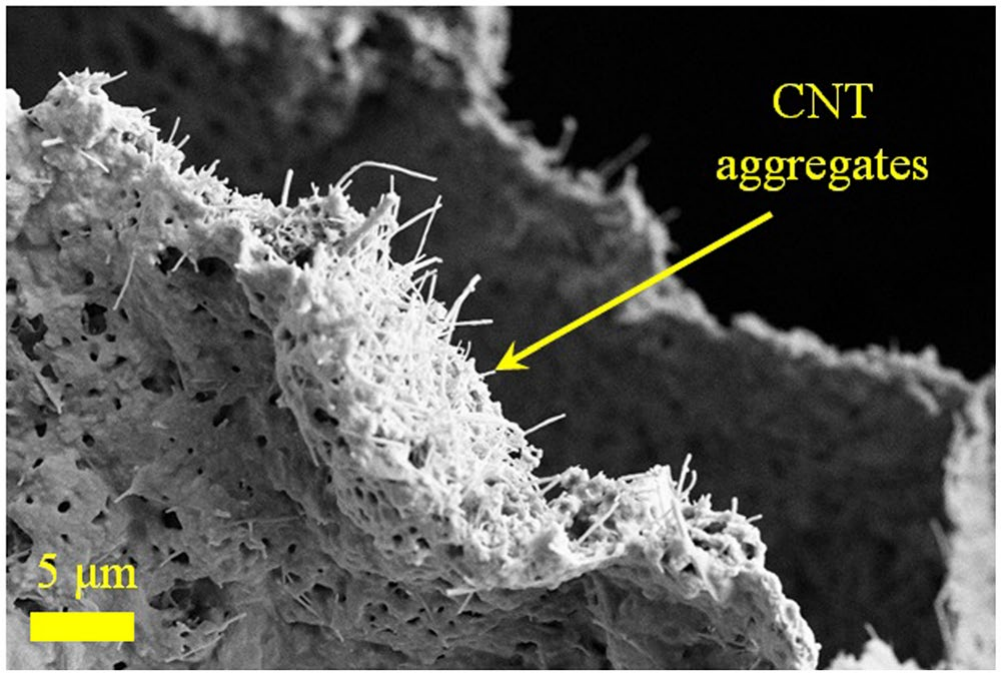
SEM micrograph of 1.5 wt% PVA-functionalized CNT at the cell walls of a sample.

In Fig. 6, SEM micrographs of a specimen with 1.5 wt% PVA-treated CNT are shown, clearly demonstrating lack of binding between the polymer and CNT. This is in sharp contrast with individual CNT adsorbed on cell walls of the 0.1 wt% CNT sample shown in Fig. 2(b). We also note that although the elastic modulus follows a similar trend as density in case of PVA-treated nanotubes, this is not the case when Triton X-100 surfactant is used for functionalization.

In the latter case, the density increases above 0.2 wt% CNT concentration, while the elastic modulus continuously reduces up to 1 wt% loading, after which it becomes constant. Interestingly, the mechanical properties at high filler concentration are quite similar for both functionalization methods, while their densities differ by 40%.

At low CNT concentrations, on the other hand, the modulus shows a strong dependence on the functionalization method. The maximum enhancement in elastic modulus compared to polymeric (non-CNT) foam with a modulus of 4.08 MPa is ~89% for the surfactant-treated CNT, while this is only 17% for samples in which the CNT are functionalized with PVA. However, at an intermediate range of 0.5–1.0% CNT concentration, the mechanical properties of PVA-treated samples are better that those of surfactant-treated ones. This is important since the covalent functionalization also yields lighter structures with higher porosity within this range of filler concentration. Therefore, there is not a one-to-one correspondence between foam density and mechanical properties, so that an optimum range can be obtained for porous composites that simultaneously provide light weight and considerable enhancement in mechanical properties *e.g*. for aerospace applications.

### Electrical conductivity

**Figure 7:**
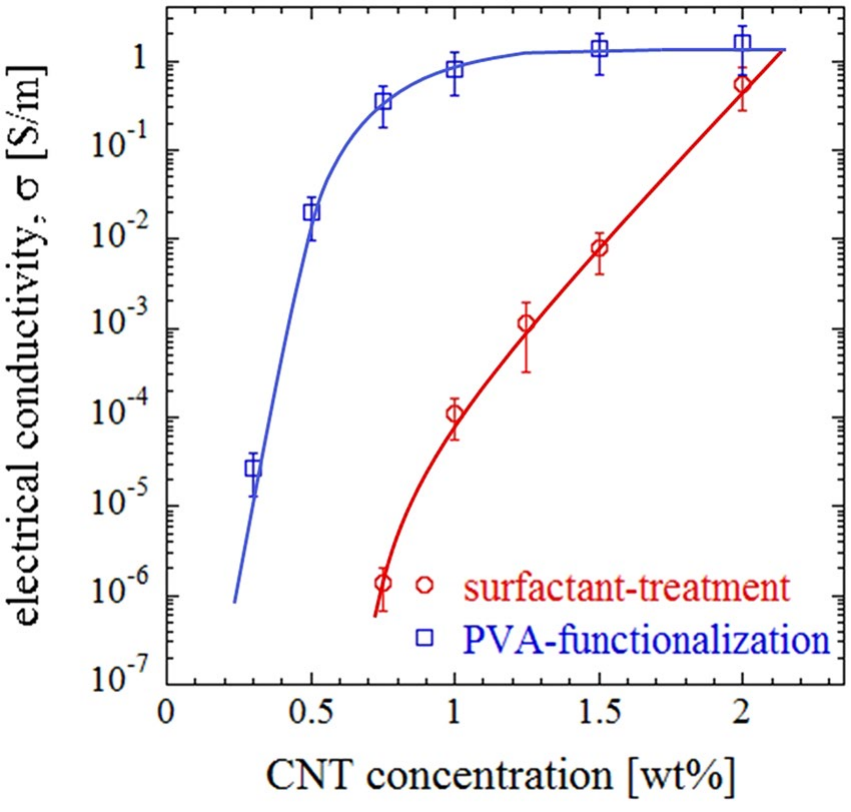
The electrical conductivity of the porous composites as a function of CNT concentration.

In order to evaluate the feasibility of proposed approach to induce electrical conductivity into porous polymers, we performed electrical measurements using a picoammeter setup. The variation of electrical conductivity with filler concentration and functionalization method is reported in Fig. 7. The conductivity curve signifies that the composite containing PVA-functionalized CNT exhibits a typical percolation behavior, with the electrical conductivity suddenly increasing several orders of magnitude above a threshold concentration. To calculate the percolation threshold, we use the statistical percolation model^59^ given by $\sigma ={\sigma }_{0}{(x-{x}_{c})}^{t}$., where *σ* is the composite conductivity, *σ*_*0*_ is the fitting coefficient expressing the conductivity of the developed network, *x* is filler concentration, *x*_*c*_ is the critical filler concentration (percolation threshold), and *t* is a dimensionality parameter of the network. Using the experimental values, the percolation threshold for PVA-treated specimens is calculated as *~0.5* *wt* *%*. A percolation threshold is not observed for surfactant-treated CNT samples. In this case, the conductivity increases almost linearly with CNT concentration over the investigated range. Note that our electrical measurements are limited by the resolution of the picoammeter setup corresponding to conductivities *σ* > *10* ^*−7*^ *S/m* for current specimens.

Additionally, we observe that at a fixed CNT content, surfactant-treated CNT samples have lower electrical conductivity. A possible explanation could be due to the interfering effect of the surfactant in foam stabilization. This may cause a higher concentration of PVDF particles to remain in the liquid film between air bubbles, which will negatively affect the construction of conductive paths by CNT in the sintered structure. This intervening role of misplaced polymer particles at a given nanofiller loading can cause incremental rather than percolative enhancement in the electrical conductivity until the plateau borders are saturated with CNT. While some studies suggest that covalent boding methods (such as PVA-functionalization) are expected to deteriorate the electrical properties of CNT possibly due to covering the CNT surface with a thin layer of the functionalizing material^43^, some other studies report their favorable effect on the electrical conductivity of CNT-polymer composites^60, 61^. In our case, this method seems to yield higher conductivities as compared to surfactant-treatment.

Comparing the conductivity of a sample containing *0.6* *wt* *%* PVA-functionalized CNT with those reported for conventional conductive polymers^18, 62–67^, we note that the electrical conductivity is within the same range at similar nanofiller/polymer loadings. Furthermore, the maximum measured conductivity is *~1.4* *S/m* at *1.5* *wt* *%* nanofiller loading, which is several orders of magnitude larger than the limit reported for other CNT-incorporated polymer composites^18, 68^. Finally, it should be noted that although PVA-functionalized CNT can yield higher electrical conductivity, the mechanical properties are inferior compared to surfactant-treatment route over a wide range of concentrations. Therefore, there exists a trade-off between optimum electrical conductivity and compressive modulus based on the target application.

### Thermal conductivity

**Figure 8:**
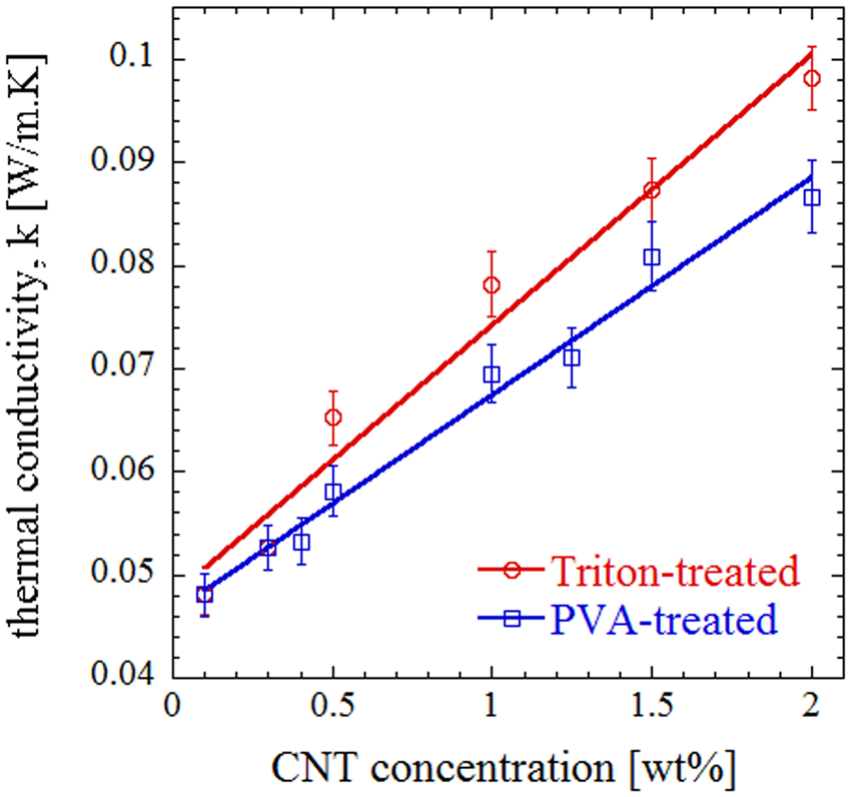
Room-temperature thermal conductivity of the porous composites as a function of CNT concentration.

In order to investigate the influence of nanofillers on thermal properties of polymer foams, thermal conductivity measurement was carried out on fabricated samples using laser flash apparatus. As shown in Fig. 8, the thermal conductivity exhibits an almost linear increase with CNT concentration for both functionalization methods. The measured conductivity seems to be independent of the functionalization method at low filler concentrations, while surfactant-treated samples show slightly higher thermal conductivity as CNT concentration increases. Comparing the calculated thermal diffusivities revealed that this is mainly due to the higher density of composite foams made of surfactant-treated CNT. Therefore, either of the two approaches could be adopted to enhance electrical or thermal conductivity based on the target application. However, the dependence of thermal conductivity to the functionalization method is negligible compared to that of the electrical conductivity. The maximum difference in thermal conductivity is ~13%, while electrical conductivity differs up to five orders of magnitude at similar CNT concentrations between the two approaches.

The increased thermal conductivity upon increasing CNT concentration in the polymer matrix is due to the intrinsically high conductivity of nanotubes^69, 70^. The thermal conductivity of polymer/CNT nanocomposites is however influenced by the large interfacial thermal resistance between the CNT and the surrounding polymer matrix, which hinders the transfer of phonon dominating heat conduction in CNT. The linear trend in thermal conductivity increase with nanotube concentration is consistent with those of reported bulk CNT/polymer composites^71–75^.

## Conclusions

A versatile approach for fabrication of electrically conductive porous polymers was developed. The procedure relies on the tendency of solid particles with appropriate size and hydrophobicity to stabilize liquid–air interfaces in aqueous foams. By tuning the wettability of a binary mixture of polymeric particles and carbon nanotubes, we could tailor their distribution in cell walls. Upon sintering the wet particle-stabilized foam, a porous composite comprised of a conductive network of nanotubes sandwiched between polymer cell walls is obtained.

We evaluated morphology, density, porosity, as well as mechanical and transport properties of the composites produced based on two different CNT functionalization methods: covalent functionalization and surfactant treatment. We find that the porous composites can reach electrical conductivities as high as *~1.4* *S/m* and densities as low as *0.13* *g/cm*3, thus making them promising candidates for light-weight applications including EMI shielding in aeronautics industry. The applicability to foam intractable polymers, the simplicity and low cost, along with the high porosity and electrical conductivity of produced composites, are among the most important features of the proposed approach.

### Data availability statement

All data generated or analyzed during this study are included in this published article.
